# Combined resection of lung cancer and thoracic aortic wall with simultaneous thoracic aortic endografting: a case report

**DOI:** 10.1186/s40792-024-01855-4

**Published:** 2024-03-08

**Authors:** Hiroaki Komatsu, Nao Furukawa, Hirotaka Kinoshita, Atsutaka Aratame, Toshio Baba, Kazunori Okabe

**Affiliations:** 1 Department of Thoracic Surgery, Bell-Land General Hospital, 500-3, Higashiyama, Naka-ku, Sakai-shi, Osaka 599-8247 Japan; 2 Department of Cardiovascular Surgery, Bell-Land General Hospital, 500-3, Higashiyama, Naka-ku, Sakai-shi, Osaka 599-8247 Japan

**Keywords:** Lung cancer, Thoracic aortic endografting, One-stage surgery, Combined resection, Thoracic aortic wall

## Abstract

**Background:**

Combined resection of lung cancer and the thoracic aortic wall with thoracic aortic endografting has been reported. However, whether the resection and endografting should be performed simultaneously or in two steps remains controversial.

**Case presentation:**

A 68-year-old man was referred to our hospital because of left chest pain. Chest contrast-enhanced computed tomography revealed a huge tumor of the left lower lung lobe, and invasion to the aortic wall was suspected. Bronchoscopic examination was performed, revealing squamous cell carcinoma with a programmed death ligand 1 expression level of 90%. The clinical stage was T4N0M0 stage 3A. After neoadjuvant chemotherapy and radiotherapy, we performed one-stage surgery with the patient in the right lateral decubitus position and the left inguinal region exposed for femoral vessel isolation. Posterolateral thoracotomy was performed with making a latissimus dorsi muscle flap. The pulmonary artery, vein, and left lower bronchus were cut with a stapler. After hilar isolation, we evaluated the involvement of the descending aorta and marked the area of the involved aortic wall by a surgical clip. Using the left femoral artery approach, a GORE TAG conformable thoracic stent graft was delivered to the descending aorta. After thoracic aortic endografting, the involved aortic wall was resected and the left lower lobe of the lung and resected aortic wall were resected en bloc. The adventitial defect was covered by the latissimus dorsi muscle flap. The operating time was 474 min, and the blood loss volume was 330 mL. The postoperative pathological diagnosis was adenocarcinoma with an epidermal growth factor receptor mutation of exon 19 deletion. The residual viable tumor was 7 mm in diameter and close to the resected aortic wall. The patient’s postoperative course was uneventful. Five days after surgery, chest contrast-enhanced computed tomography revealed no endoleak or stent migration. Three months after surgery, he was alive with neither recurrence nor stent graft-related complications.

**Conclusions:**

One-stage surgery involving combined resection of lung cancer and the thoracic aortic wall with simultaneous thoracic aortic endografting in the right lateral decubitus position with the left inguinal region exposed is safe and acceptable.

## Background

Combined resection of lung cancer and the thoracic aortic wall with thoracic aortic endografting has been reported in selected patients [1–5]. The most suitable therapy for locally advanced T4 lung cancer varies and may include combinations of surgery, chemotherapy, immune checkpoint inhibitors, and radiotherapy. Moreover, whether the resection of lung cancer involving the thoracic aortic wall and thoracic aortic endografting should be performed simultaneously or in two steps remains controversial. We herein report a case involving a patient who successfully underwent combined resection of lung cancer and the thoracic aortic wall with simultaneous thoracic aortic endografting 1 month after neoadjuvant chemoradiotherapy.

## Case presentation

**Fig. 1:**
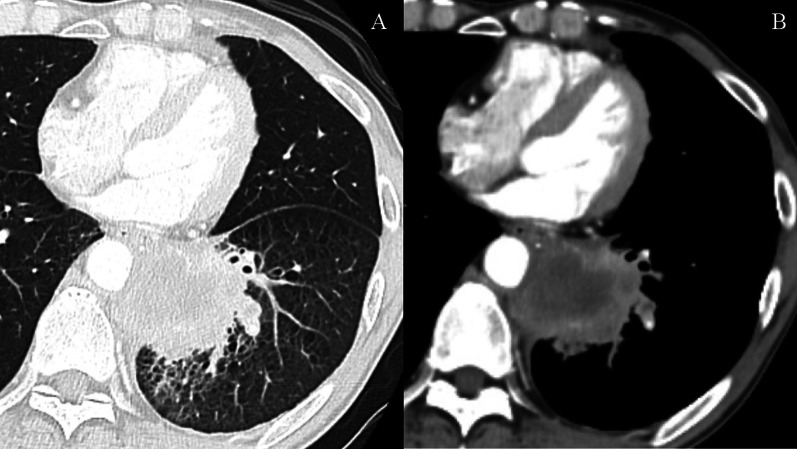
Chest contrast-enhanced CT before neoadjuvant chemoradiotherapy. Chest contrast-enhanced CT showing a huge tumor (diameter; 7 cm) of the left lower lobe of the lung. The tumor was close to the descending aorta, and the invasion to the aortic wall was suspected. CT: computed tomography

**Fig. 2:**
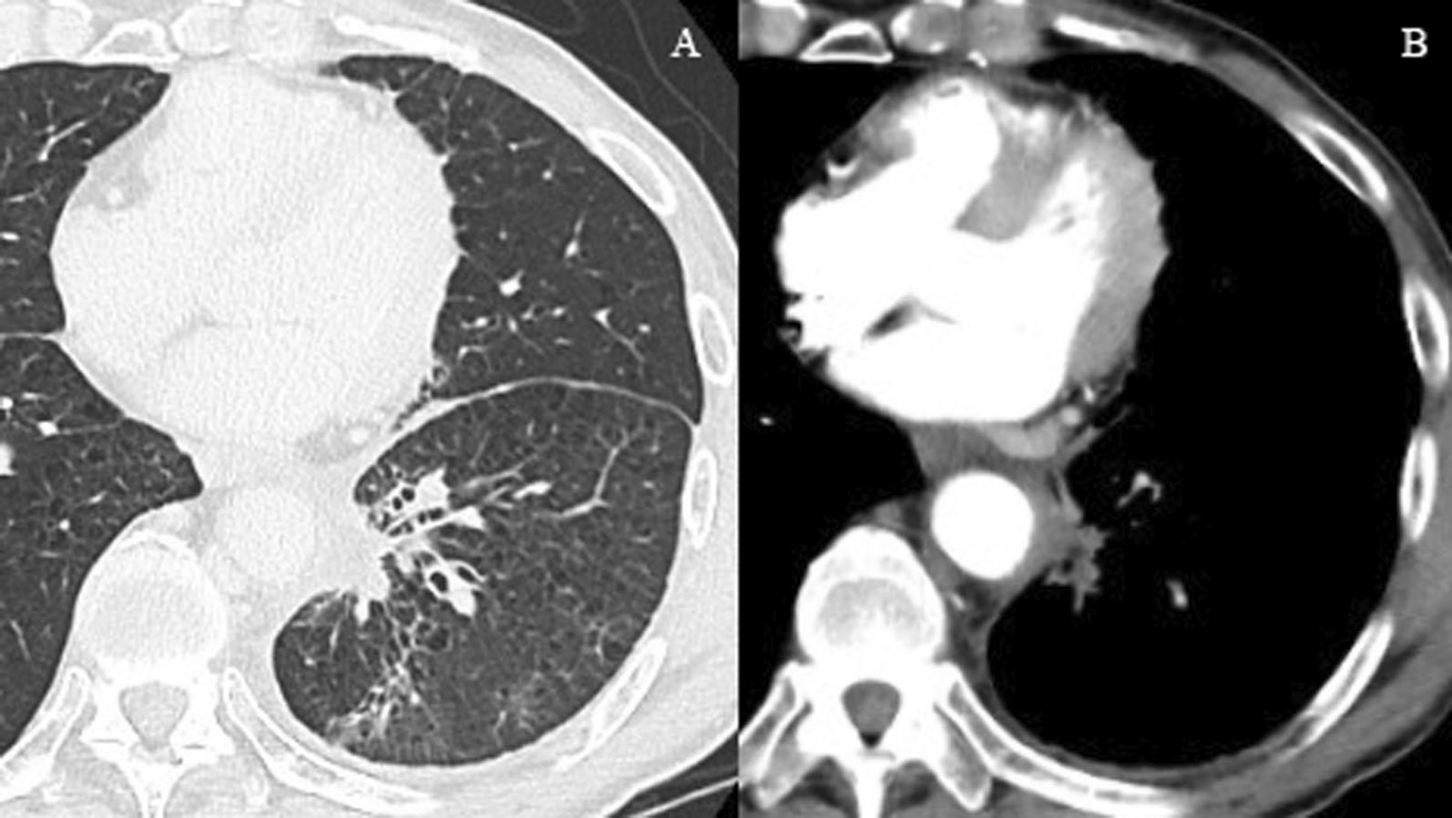
Chest contrast-enhanced CT after neoadjuvant chemoradiotherapy. One month after the chemoradiotherapy, chest contrast enhanced CT showing even reduction of the tumor. CT: computed tomography

**Fig. 3:**
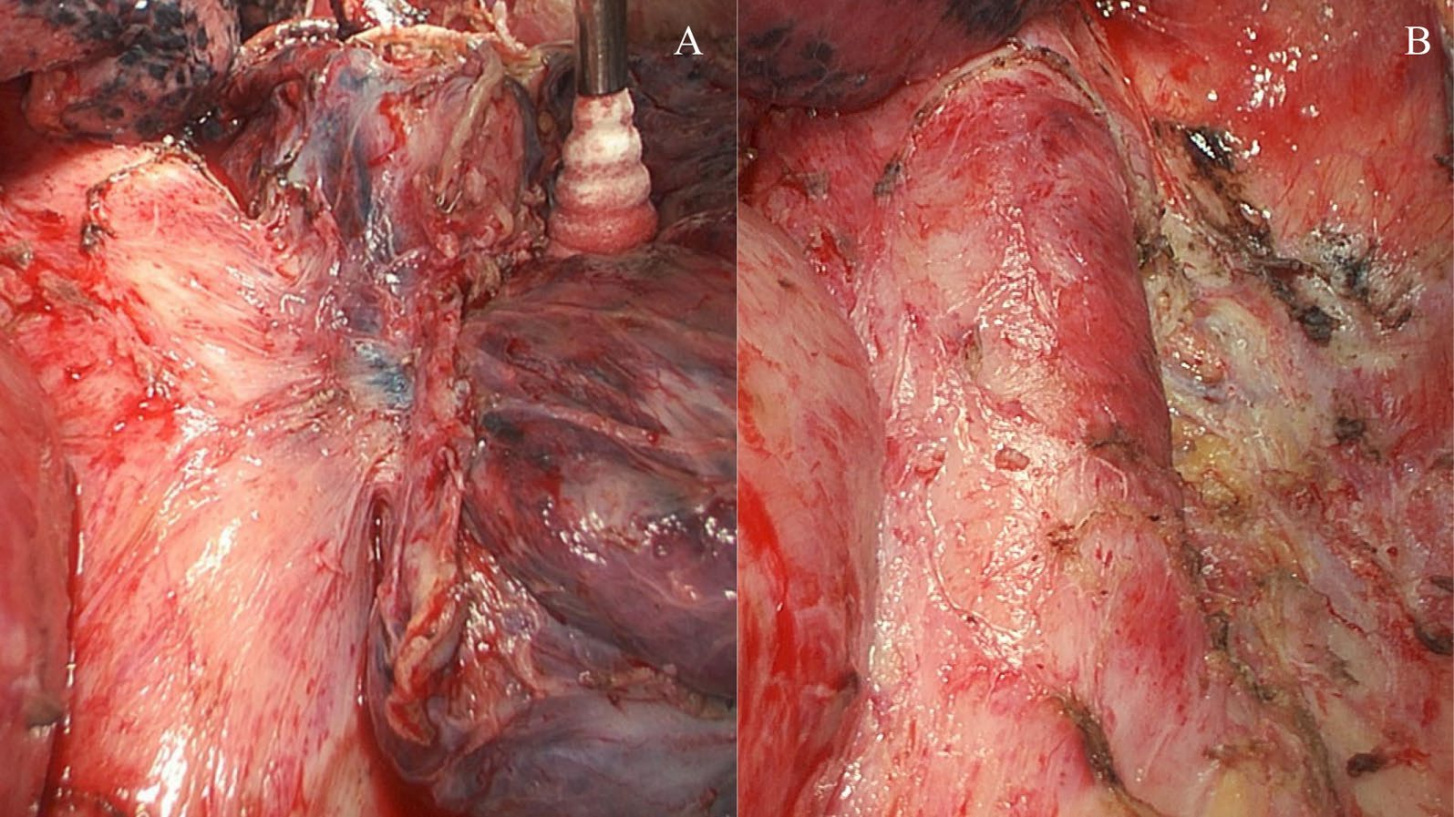
Surgical view of the involved descending aorta. **A** Surgical view showing the involvement of the tumor to the descending aorta. **B** Surgical view after combined resection of the aortic wall showing the defect of one-half of the adventitia. There was no exposure of the stent graft

**Fig. 4:**
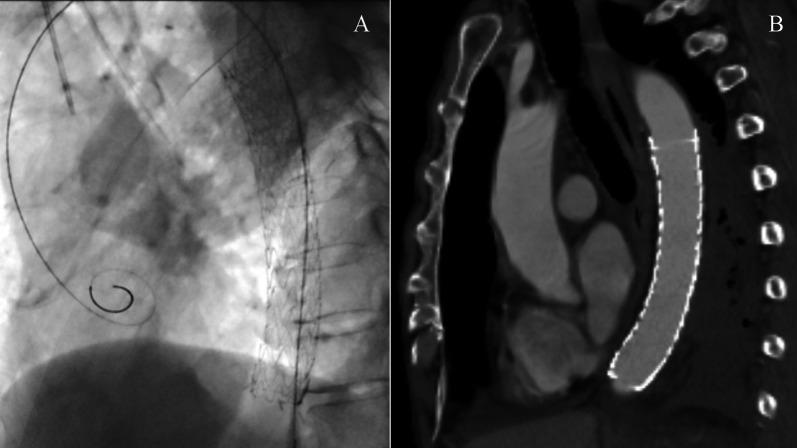
Chest X-ray and contrast-enhanced CT of the stent graft. **A** Chest X-ray showing the stent graft delivered to the descending aorta. **B** Five days after surgery, chest contrast-enhanced CT revealed no endoleak or stent migration. CT: computed tomography

**Fig. 5:**
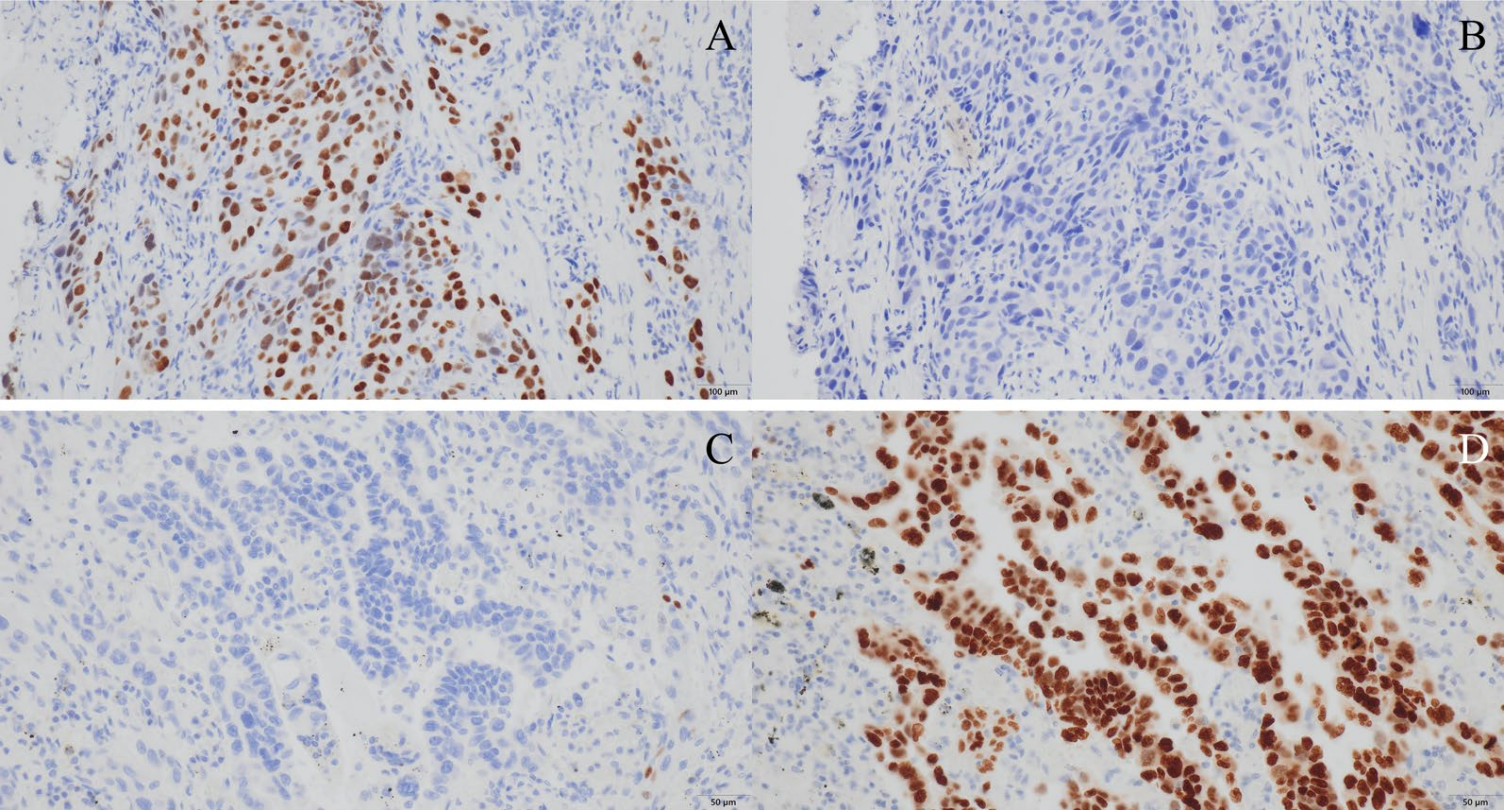
Pathological examination before and after the surgery. Preoperative pathological examination showed squamous cell carcinoma with **A** positive expression of p40 and **B** negative expression of TTF-1 by immunohistochemistry. Postoperative pathological examination showed adenocarcinoma with **C** negative expression of p40 and **D** positive expression of TTF-1 by immunohistochemistry. TTF-1, thyroid transcription factor 1

A 68-year-old man was referred to our hospital because of left chest pain. Chest contrast-enhanced computed tomography (CT) revealed a huge tumor (diameter of 7 cm) of the left lower lung lobe (Fig. 1). The tumor was close to the descending aorta, and invasion to the aortic wall was suspected. Positron emission tomography revealed no distant metastasis or lymph node metastases. Bronchoscopic examination was performed, revealing squamous cell carcinoma with positive expression of p40 and negative expression of thyroid transcription factor 1 by immunohistochemistry (Fig. 5A, B). The programmed death ligand 1 (PD-L1) expression level was 90%. The clinical stage was T4N0M0 stage 3A. We planned neoadjuvant chemoradiotherapy followed by left lower lobectomy and aortic wall resection with thoracic aortic endografting. First, chemotherapy (carboplatin and vinorelbine) and radiotherapy (40 Gy) were performed. One month after the chemoradiotherapy, chest contrast-enhanced CT revealed even reduction of the tumor (Fig. 2). We then performed one-stage surgery with the patient in the right lateral decubitus position with the left inguinal region exposed for femoral vessel isolation. Posterolateral thoracotomy was performed with making a latissimus dorsi muscle flap. Widespread adhesions between the left lung and the thoracic wall were observed, and all adhesions were dissected. The adhesion of the left lung to the mediastinal side was particularly severe and was dissected carefully. About 2 h were required to dissect the severe intrathoracic adhesions. We first dissected the esophagus and inferior pulmonary vein, confirming no involvement. We then concluded that the complete resection by left lower lobectomy and aortic wall resection with thoracic aortic endografting was acceptable. The pulmonary artery, pulmonary vein, and left lower bronchus were cut with a stapler. After hilar isolation, we evaluated the involvement of the descending aorta (Fig. 3A) and decided to resect the aortic wall. We marked the area of the involved aortic wall by a surgical clip. Using the left femoral artery approach, a GORE TAG conformable thoracic stent graft was delivered to the descending aorta according to the marked surgical clip (Fig. 4A). It took 41 min to deliver the stent graft. After thoracic aortic endografting, the involved aortic wall was resected (Fig. 3B), and the left lower lobe of the lung and resected aortic wall were resected en bloc. One-half of the adventitia of the aortic wall was resected. There was no exposure of the stent graft, and the defect of the adventitia was covered by the latissimus dorsi muscle flap. The operating time was 474 min, and the blood loss volume was 330 mL. The postoperative pathological diagnosis was adenocarcinoma with positive expression of thyroid transcription factor 1 and negative expression of p40 by immunohistochemistry (Fig. 5C, D). Additionally, epidermal growth factor receptor (EGFR) mutation of exon 19 deletion was present. Because of the severe fibrosis around the aortic wall after preoperative treatment, the structure of the vessel wall was unclear and the invasion to the aortic wall could not be evaluated. The residual viable tumor was 7 mm in diameter and close to the resected aortic wall. The patient’s postoperative course was uneventful. Five days after surgery, chest contrast-enhanced CT revealed no endoleak or stent migration (Fig. 4B). He was discharged 19 days after the surgery. Three months after the surgery, he was alive with neither recurrence nor stent graft-related complications.

## Discussion

Both one- and two-stage combined resection of lung cancer and the thoracic aortic wall with thoracic aortic endografting have been reported [1–5]. Sato et al. [2] reported a one-stage surgery in which the patient’s position needed to be changed twice. First, in the lateral position, the infiltrated aortic wall was confirmed and marked with a surgical clip. Second, in the supine position, endovascular stent grafting was performed. Third, in the lateral position, the lung cancer and involved aortic wall were resected en bloc. Di Tommaso et al. [3] also reported a single-stage procedure with stent grafting in the supine position. In our patient, the tumor invaded to the only descending aorta, and the sufficient landing zone of the stent graft was expected. As in a previous report by Marulli et al. [1], we performed one-stage surgery without changing the patient’s position because endovascular stent grafting to the descending aorta could be performed safely in the right lateral decubitus position with the left inguinal region exposed. One-stage surgery has some advantages with respect to the surgical indication and methods. One-stage surgery can facilitate adequate evaluation of the area of aortic wall in which the lung cancer is involved and the stent grafting is needed. Thoracoscopic examination cannot reveal how the tumor involves the aortic wall, especially when wide adhesions are present in the thoracic cavity, as in our patient. When the aorta is not involved, unnecessary aortic endografting should be avoided because of the risk of stroke or paraplegia. Few reports have described successful pulmonary resection with combined resection of both the esophagus and aortic wall, suggesting that patients with involvement of both the esophagus and aortic wall have an even poorer prognosis and surgical outcome. When performing pulmonary resection with combined resection of both the esophagus and aortic wall, many fatal complications can occur, such as impaired blood flow of the bronchial stump or esophagus, bronchopleural fistula, anastomotic leakage of the esophagus, and infection of the aortic stent graft or synthetic graft. Some of these complications may be inoperable. At least a two-stage surgery is recommended, and the esophagus should be subsequently reconstructed [6]. In our patient, we found no involvement of the esophagus before cutting the pulmonary vessels or performing the aortic endografting, and we determined that we would be able to resect the entire tumor by one-stage surgery. Unnecessary intraoperative aortic endografting can also be avoided in one-stage surgery. The disadvantages of one-stage surgery are the instability of the stent graft and the risk of endoleak formation. No reports have described stent graft-related complications, including paraplegia, stent migration, endoleaks, or bleeding, in one-stage surgery [1–3]. Two-stage surgery enables clinical and radiological assessment of these stent graft-related complications before the resection [5]. Five days after surgery in our patient, chest contrast-enhanced CT revealed no endoleak or stent migration. When left lower lobectomy with stent grafting of the descending aorta is performed, we believe that our surgical technique and patient positioning are safe and acceptable. However, most patients in previous reports underwent pulmonary resection several days after aortic endografting in a two-stage surgery [2–5]. Further studies involving larger sample sizes are needed to evaluate the outcomes of one-stage surgery.

It has been reported that aortic wall buttressing by a synthetic patch or omental flap should be considered when more than one-half of the circumference or the full thickness of the aortic wall is resected [1]. In our patient, one-half of the circumference of the adventitia was resected, and we avoided reinforcement of the aortic wall defect by a synthetic patch considering the risk of postoperative infection. At the thoracotomy site, we did not cut the latissimus dorsi muscle; we made a flap of the latissimus dorsi muscle, which was then used to cover the defect of the adventitia of the aortic wall. Collaud et al. [5] reported that a large aortic defect was buttressed with bovine pericardium and a latissimus dorsi muscle flap that had been preserved at the time of thoracotomy. We believe the latissimus dorsi muscle flap is less invasive than the omental flap for aortic wall buttressing, and preservation of the latissimus dorsi muscle should be considered at the time of thoracotomy. When the full thickness of the aortic wall was resected, the aortic wall buttressing by a synthetic patch should be considered in addition to the latissimus dorsi muscle flap. Stent graft herniation through the aortic wall has been reported [7], and long-term follow-up is needed to evaluate not only the recurrence of lung cancer but also any aortic-related complications.

Interestingly, preoperative histology revealed squamous cell carcinoma, whereas postoperative histology revealed adenocarcinoma. This suggests that the squamous cell carcinoma component disappeared with chemoradiotherapy and that the adenocarcinoma component remained. The residual tumor was located close to the resected aortic wall, and all the tumor tissue was completely resected by combined resection of the aortic wall. Neoadjuvant chemotherapy and immune checkpoint inhibitor therapy were recently recommended for clinical stage 2 and 3 cancer [8]. However, whether chemoradiotherapy or immune checkpoint inhibitor therapy is suitable as preoperative therapy for locally advanced T4 lung cancer is controversial. Selection of the most appropriate therapeutic strategy is based on the histological type, driver gene alterations involving EGFR and anaplastic lymphoma kinase rearrangements, or expression of PD-L1. Our patient had both squamous cell carcinoma with high PD-L1 expression and adenocarcinoma with EGFR mutation of exon 19 deletion, and he successfully underwent complete resection by the combination of chemoradiotherapy and surgery. An immune checkpoint inhibitor or EGFR tyrosine kinase inhibitor will probably be effective if postoperative recurrence occurs in the future, and long-term survival can be expected in our patient. In patients with T4 lung cancer showing high PD-L1 expression, neoadjuvant immune checkpoint inhibitor therapy might be more effective and induce less inflammatory change than chemoradiotherapy. Our patient had severe adhesions and inflammatory change, which required careful dissection and a longer operating time. Neoadjuvant immune checkpoint inhibitor therapy might make the surgery easier to perform than neoadjuvant chemoradiotherapy because of the decrease in inflammation and fibrosis after radiotherapy. A prospective study is needed to evaluate the preoperative local control of T4 lung cancer by comparison of chemoradiotherapy and immune checkpoint inhibitor therapy.

## Conclusion

One-stage surgery involving combined resection of lung cancer and the thoracic aortic wall with simultaneous thoracic aortic endografting in the right lateral decubitus position with the left inguinal region exposed is safe and acceptable.

## Data Availability

All data are available upon reasonable request.

## Abbreviations

CT: Computed tomography
PD-L1: Programmed death ligand 1
EGFR: Epidermal growth factor receptor

## Availability of data and materials

Applicable.
